# Introduction

**DOI:** 10.1007/978-3-030-54895-7_1

**Published:** 2020-12-10

**Authors:** Stephany Griffith-Jones, Bettina De Souza Guilherme

**Affiliations:** 1 Founder and Co-coordinator of the JeanMonnet Network “Crisis-Equity-Democracy for Europe and Latin America”, IRELAC, Rio de Janeiro, Brazil; 2 Founder and Co-coordinator of the JeanMonnet Network “Crisis-Equity-Democracy for Europe and Latin America”, IRELAC, Rio de Janeiro, Brazil; 3 Initiative for Policy Dialogue, Columbia University, New York, USA; 4 Pontifical Catholic University of Rio de Janeiro (PUC-Rio), Rio de Janeiro, Brazil; 5 Initiative for Policy Dialogue, Columbia University, New York, USA; 6 Institute of Development Studies, Sussex University Brighton, Brighton, UK; 7 Founder and Co-coordinator of the Jean Monnet Network “Crisis-Equity-Democracy for Europe and Latin America”, IRELAC, Rio de Janeiro, Brazil; 8 European Parliament, Brussels, Belgium

## Abstract

This book is the result of the first 3 years of the comparative and multidisciplinary Jean Monnet Network, “Crisis-Equity-Democracy for Europe and Latin America”, of senior academics and policy advisors from four European and three Latin American countries, including experts on the European Union and Latin American regionalism. The rationale of the project and the common link is that both Europe and Latin America can learn from their respective experiences on “crisis”, its management and the distributive and democratic implications at national and regional level. The main purposes of the joint research can be summarised as to (1) locate in the current global financial system as one of the very major causes of the financial and debt crises in the EU and Latin America; (2) demonstrate the impact of the paradigm change on global and EU economic governance; (3) analyse key systemic aspects of the global crisis, i.e. climate change, macro-financial instability and the weakening of democracy and their inter-connections; (4) map and evaluate how both regions and individual countries within both regions have tried to manage these crises; (5) discuss the economic, political and social effects of these crises on both regions and individual countries; (6) finally, to make policy suggestions on how to transition from finance capitalism to a more sustainable real capitalism, on how both regions can better manage/govern/respond to such systemic pressures and on how they can increase their cooperation.

This book is the result of the 3 years comparative and multidisciplinary Jean Monnet Network, “Crisis-Equity-Democracy for Europe and Latin America”, of senior academics and policy advisors from four European and three Latin American countries, including experts on the European Union and Latin American regionalism.

The rationale of the project and the common link is that both Europe and Latin America can learn from their respective experiences on “crisis”, its management and the distributive and democratic implications at national and regional level. The Latin American debt crisis of the 1980s and the global financial crisis of 2008 and the following Eurozone debt crisis have much in common in regard to their economic and social impacts and the following wave of populism and polarisation. Furthermore, the research and exchange in the network clearly showed that these crises are symptoms and consequences of the same systemic flaws inherent both in the present EU and global financial, economic and governance system. Given that both regions face fairly similar global unsustainability challenges, from a macro-financial as well as an environmental point of view, their similarities should call for the joint elaboration and implementation of a bi-regional strategic alliance for addressing the roots of the global crisis.

The main purposes of the project can be summarised as to (1) locate in the current global financial system one of the very major causes of the financial and debt crises in the EU and Latin America; (2) map and evaluate how both regions and individual countries within both regions have tried to manage these crises; (3) discuss the economic, political and social effects of theses crises on both regions and individual countries; (4) and, finally, to make policy suggestions on how to transition from finance capitalism to a more sustainable real capitalism, on how both regions can better manage/govern/respond to such systemic pressures and on how they can increase their cooperation.

The book begins in Chap. 10.1007/978-3-030-54895-7_1 with a theoretical introduction which includes a chapter by Stephan Schulmeister that explores the effects of the paradigm change on economic policies in Europe and in Latin America and a second chapter by Christian Ghymers, which analyses some key systemic aspects of the global crisis, i.e. climate change, macro-financial instability and the weakening of democracy and upon their inter-connections. These intertwined aspects reflect myopic prices, the non-incorporation of externalities as well as the lack of needed regulation of financial markets and are also linked by the way public decisions are biased towards the short-term, and influenced by vested interests.

The following chapters are divided in five parts. The second part explores aspects of regional governance and how the economic and financial crises were managed in Europe and Latin America. The chapters share a common diagnosis that, with the breakup of the Bretton Woods System and the following liberalisation of capital flows and deregulation of financial markets, the emerging countries have been subject to bubbles and crisis, in which they were confronted with capital flight to safe havens similar to what occurred later to the countries in the euro area most affected by the crisis and in both cases their central banks did not have the control over the currency in which their debts were denominated. Paul De Grauwe, for instance, compares the situation of a debtor country within the Eurozone to the status of the emerging countries in a debt crisis and reveals similarities because “they cease to have control over the currency in which their debt is issued. As a result, financial markets can force these countries’ sovereign defaults. This makes the monetary union fragile and vulnerable to changing market sentiments” (De Grauwe 2012).

In both regions, debtor countries had at most insufficient (public) debt relief but suffered major (internal) devaluations (lowering of real wages and pensions and cuts in essential public services, especially health and education) and a prolonged period of economic stagnation, decline or low growth. The IMF adjustment programmes for Latin America and the Troika programmes for Europe’s debt crisis countries bear indeed strong similarities and economic and social consequences leading to a lost decade, decline of the middle class, lowering of living standards of poorest people and fall in investment, the increase of inequality and poverty by reducing the role of the state, cuts in public services and social spending. Griffith Jones identifies the vulnerability of European creditor banks to debt reduction as a key problem in the euro area debt crisis, as it was in the Latin American debt crisis, which was managed in the interests of the creditor banks and countries: this is in one sense much more serious in the European case than in Latin America, because the main lender to Southern European countries were creditors and investors from other European countries. This and major flaws in the architecture of EMU as well as their lack of accountability played against an EMU crisis management based on the “general interest” of the European Union and are a major cause for the publicly displayed conflict between debtor and creditor countries leading to a nearly existential crisis of Economic and Monetary Union, much to the detriment to the European Union’s image within the European Union, in Latin America and globally.

Comparing the crises of the 1980s, 1990s and 2010s, we can identify failures of the coordination of both macroeconomic policies and the absence of debt restructuring mechanisms at international level (both at EU and at the global level). Failures in providing these coordination efforts could entail, as was the case in the 1980s, and is again in the 2010s, a delay in implementing the required solutions making the social cost of these episodes more profound (Sanguinetti 2014).^1^

There are, however, differences in timing and sequence of events, in the strength of the “social model”, and concerning the process of regionalism. Latin America has been a pre-runner or prototype of debt crises, but both the social model and regionalism are much more developed in Europe. In fact, in Europe, where both debtor and creditor countries were in the same economic area, the European Union has been both part of the problem as a trigger of the crisis and key to solutions of the crisis. In Latin America, regionalism never achieved the same level of integration, nor was there a serious ambition or project that could pair the EU. At the time of the 1980s Latin American debt crisis, there was an attempt to coordinate different Latin American debtor positions (in the ad hoc Cartagena Group), but it had limited impact, due to insufficient political commitment by the Latin American debtor governments and effective divisive tactics by the creditor governments. Furthermore, creditors were outside Latin America. Moreover, regional organisations have been much more of a political nature in Latin America. Except for the recent results of the Pacific Alliance, even the organisations, which prioritised the liberalisation of trade such as Mercosur, never achieved full free trade. Additionally, due to the increasing political polarisation in the last years, the existing regional organisations even lost the capacity to effectively intervene in the cases of (political) crisis to mediate a solution.

The volume takes account of these differences by having a strong focus on the analysis of the architectural flaws and the democratic deficit of the Economic and Monetary Union and analysing and proposing a number of reforms of EMU, ranging from Treaty reform to reforms not requiring Treaty reforms. It presents proposals on policy priorities, including development and strategic partnerships promoting the green transition, greater cooperation for SMEs and a macroeconomic cooperation between the two regions in the framework of a renewed strategic alliance. The proposals thus foresee both the reforms to fix the problems, which triggered the crisis, and proposals, which lead the way out of the crisis and into a more sustainable future for both regions with the EU as a motor.

On Latin America, we address the issue of regionalism in three chapters: one on the structure of crisis management at regional level, a second one on the impact of crisis on regionalism in the region and the third on the structural lack of total factor productivity increases, which includes proposals for improving it including by a strategic alliance with the EU. The second one deals mainly with the impact of the crisis in some of the regional key players on regionalism and the effectiveness of regional organisations addressing crises. Furthermore, it is obvious that the failure of an adequate crisis management based on solidarity at European level, the application and enforcement of similar adjustment programmes as in Latin America in spite of Europe’s pride in its social model and social market economy, had the consequence of undermining confidence and enthusiasm for the European Union.

The third and fourth parts explore the impact of the crisis in Europe and Latin America, at the national and regional levels, including the political polarisation and rise in populism. The crisis in Latin American countries follows a different timeline, and the developments in Latin America seem to be ahead of Europe. In the aftermath and possibly as a reaction of the lost decade of the 1980s and the intensive austerity programmes, most of the key countries in Latin America experienced a “pink wave” of more left-wing and/or populist governments, followed by a “blue wave of neoliberalism”. The interesting point in common between the two regions is that after the crisis and the austerity era, political positions, parties and governments seem to be more polarised and less stable. Some of the European populist parties on the left, such as Podemos or Syriza, were to some extent taking these movements and their strategies as a model. However, in Europe, left-wing parties and other organisations were not able to benefit from the “systemic crisis of neoliberal capitalism” to use the momentum to change both European and Global governance, while the “pink wave” did lead to establishment of a number of regional organisations and the rise at least of Brazil as one of the BRICS and motor of South-South and sustainable development for a certain period, i.e. until about 2013.

During this period, in Europe, the financial crisis and the following and overlapping migration crisis with an equally “poor” crisis management and lacking solidarity by the European Union both furthered the rise of right-wing populism, xenophobia, nationalism and euro-scepticism jeopardising liberal democracy, regional integration and multilateralism. This poses an obstacle to put into place the necessary reforms in the design of the European Union to remedy the revealed flaws of EMU’s architecture and keeps the inbuilt weaknesses. A re-nationalisation of political competences will rather further increase the vulnerability in a system of global financial markets without adequate political and democratic governance and regulation. The current political wave is one of a retreat into national populism and putting into question regionalism, the urgency of climate change and multilateralism.

Given the rise of nationalism, soveranism and populism, Part IV contains five articles on these topics and their impact on regionalism, including a chapter on the populism in Europe. Additionally, a number of case studies in Part III, such as Greece, Italy, Portugal, Venezuela and Brazil, have integrated the rise of populism within the analysis.

While the starting point in both regions are financial crises caused by deregulated financial markets in the aftermath of the break-up of the Bretton Woods system, the research also analyses the governance and legitimacy crisis regarding the crisis management and economic, social and political consequences at EU level and at national level in a sample of the debtor countries. Greece was chosen for not only being a highly indebted country at the moment the crisis broke out (in fact triggering the Eurozone debt crisis) but also having been submitted to intentionally tough and punitive austerity programmes, with extremely negative effects on the economy and the majority of its people, which were clearly and repetitively rejected by its population. Additionally, the Greek sovereign debt crisis led to a nearly existential crisis of the euro area through the risk of contagion. Portugal had been suffering from a lack of competitiveness. While the first (right wing) government rather outperformed the Troika adjustment programmes to implement pro-market structural reforms, the electorate as a consequence exchanged the government for a left-wing coalition which turned the Portuguese programmes into a success story of a country undergoing adjustment programmes, with a high degree of ownership taking into account the policy choices of the population, and with a more pro-Keynesian macroeconomic policy, that yielded positive results.

Italy went through an economic and political crisis triggered by the sovereign debt crisis and amplified by the incomplete structure of the Eurozone. The ingrained causes were the long-term structural problems and high public debt levels, leaving it vulnerable to external shocks. Italy did not undergo a Troika-led adjustment programme; however such adjustment was imposed by a technocratic government implementing austerity measures and reforms to signal to markets and stop the spiral of distrust and negative self-fulfilling expectations, but in fact so far not (successfully) addressing the sources of the problems, in particular the lack of total factor productivity growth. Italy is the example for a bigger and more powerful crisis country in which the disaffected voters turned for a time to right-wing parties, which temporarily became part of the government and confronted the other European partners by putting into question the existing rules of Economic and Monetary Union.

For the case studies in the region of Latin America, we have selected a number of key players undergoing major crisis with economic and political consequences for the entire region: Brazil, which had developed into the regional leader of South America, and Venezuela, which promoted and actively supported a populist and ideological anti-market regionalism. Brazil and Venezuela are addressed in a more in-depth analysis which focuses predominantly on the contemporary (political) crisis, including the rise of polarisation and populism, although it is clear that economic development and political crisis constantly feed on each other and can never be regarded as completely independent. Another interesting aspect we address in our volume is the rise and role of new social movements and new political parties both in Latin America and in Europe. In Venezuela and Brazil, we can observe the mobilisation of major social movements and protests as a turning point of the “pink tide”, the left-wing governments, with the subsequent loss of public support, leading in both cases to a regression of the democratic development. In both cases, the economic and political crisis is partly related to the “resource curse”, with an over-dependence on petrol and other primary resources and major corruption scandals and the decline of the economy in line with the fall of petrol prices. In the case of Venezuela, the mixture of the economic mismanagement and even humanitarian crisis, the political crisis and the foreign political tensions with the USA led to the conversion of the government into a left-wing authoritarian regime. In Brazil, the economic slowdown was used as a window of opportunity to impeach the elected president accusing her of creative accounting and reversing the left-wing government agenda into a Washington Consensus programme. At the following elections, the most popular (left wing) candidate was impeded to stand for elections by a judicial-political intrigue, paving the way for a populist authoritarian regime from the extreme right.

The crisis has also recently led to violent events even in successful economies in Latin America, like Chile, a country which in spite of very impressive growth (till recent years) and poverty reduction achievements had not only a very unequal income distribution but also still a rather extreme version of the market economy, in aspects like pension system, health and education. Therefore, this country also faces major challenges not just to restore growth but to significantly improve income distribution, as well as make important reforms in its economic development model, especially in sectors like its pension, health and education systems away from an extreme market economy, to a more balanced model, that involves a greater role of government institutions to deliver public goods, like good health and education at reasonable cost for all, including the poorer segments of the population, as well as sufficient pensions for the relatively poorer people, via solidarity redistributive mechanisms. Moreover, we also analyse the impact of their crisis on regional institutions in Latin America and regional integration. The book furthermore has a comparative study on the economic policies of Argentina, Brazil and Mexico.

One of the main conclusions of the network and project that is both valid for Europe and for Latin America is that the current financial system provokes economic, social, ecological and political instability and urgently needs to be reformed.

The last chapters, in Part VI, present proposals of the network reaching from the transition from finance capitalism to an economically, socially and ecologically sustainable real capitalism, to reforms in both regions and at the inter-regional level of EU-LAC relations. The increase of inequality in some countries and weakening of the middle classes as well as insufficient and slowing down of income increase of the poorer segments in recent years led to the loss of trust in the traditional political parties, often captured by the financial markets, an increased polarisation and the rise of populist forces, parties and political leaders. To give an answer to these developments, and in order to introduce more “stability”, “sustainability” and “equity” into the current European and global model, Europe has the potential and the responsibility to develop a European “Eco-social model” as a prototype and motor for a global “Eco-social model”. The book introduces the potential of modernising and enlarging the welfare state as probably the most important fundament of a new/renewed “European identity”. New labour time models as prerequisite for continuing economic growth with continuous implementation of technical progress (raising labour productivity) and sustained full employment are desirable aims. An active modern industrial policy, partly funded by public development banks, which can help redirect also private finance to key sectors, is also essential to make growth sustainable, inclusive and dynamic.

The network considers the bi-regional EU-LAC cooperation as a further stepping stone in stimulating and reviving multilateralism and improving global governance through a “macroeconomic dialogue” between academics, civil society and officials, a “public-private strategy with a view to improve the joint competitiveness and the participation in GVCs in the global markets” as well as sustainable investment projects promoted via the EIB, targeted to achieve the new “green and social deal” for Europe, Latin America and globally. A more intensive cooperation of the two regions could be not only be mutually beneficial in times of major trade tensions but especially represent a valuable contribution to overcome the current crisis of multilateralism and global governance. The COVID-19 pandemic crisis, which evolved after the end of the research conducted by the authors of this book reinforces the arguments put forward, and the necessity to deepen the cooperation between the European Union and Latin America. We have also received the good news about the renewal of the Jean Monnet Network “Crisis-Equity-Democracy for Europe and Latin America”, what will enable the researchers to continue their join efforts to reflect upon the crises, and advance proposals to the regions and their political dialogue, contributing to foster EU-CELAC Strategic Alliance.

## Footnotes

1 Sanguinetti Pablo, Comments on “The Latin American Debt Crisis in Historical Perspective” by José Antonio Ocampo in Stiglitz, Joseph E. (2014-07-23). Life After Debt: The Origins and Resolutions of Debt Crisis (International Economic Association) (Kindle Locations 2943–2944). Palgrave Macmillan. Kindle Edition.
